# Two novel mitoviruses from a Canadian isolate of the Dutch elm pathogen *Ophiostoma novo-ulmi* (93–1224)

**DOI:** 10.1186/1743-422X-10-252

**Published:** 2013-08-08

**Authors:** William E Hintz, Joyce S Carneiro, Irina Kassatenko, Aniko Varga, Delano James

**Affiliations:** 1 Department of Biology, University of Victoria, P.O. Box 3020 STN CSC, Victoria, BC V8W 3N5, Canada; 2 Canadian Food Inspection Agency, 8801 East Saanich Road, Sidney, BC V8L 1H3, Canada

**Keywords:** *Ophiostoma novo-ulmi*, dsRNA virus, Hypovirulence, Mitovirus, Dutch elm disease, Biological control

## Abstract

**Background:**

*Ophiostoma novo-ulmi* is the causative agent of Dutch elm disease (DED). It is an ascomycetous filamentous fungus that ranks as the third most devastating fungal pathogen in Canada. The disease front has spread eastward and westward from the epicentre in Ontario and Quebec and is threatening elm populations across the country. Numerous mitigation strategies have been tried to eradicate this pathogen, but success has thus far been limited. An alternative approach might utilize double-stranded RNA (dsRNA) mycoviruses which have been reported to induce hypovirulence in other fungi.

**Methods:**

Using a modified single primer amplification technique (SPAT) in combination with chromosomal walking, we have determined the genome sequence of two RdRp encoding dsRNA viruses from an *O. novo-ulmi* isolate (93–1224) collected from the disease front in Winnipeg.

**Results:**

We propose that these viruses, which we have named OnuMV1c and OnuMV7 based on sequence similarity to other *Ophiostoma* mitoviruses, are two new members of the genus *Mitovirus* in the family Narnaviridae.

**Conclusions:**

The discovery of such dsRNA elements raises the potential for engineering these viruses to include other genetic elements, such as anti-sense or interfering RNAs, to create novel and highly specific biological controls. Naïve fungal hosts could be infected with both the engineered molecule and a helper mitovirus encoding an RdRp which would provide replication capacity for both molecules.

## Background

Natural and urban populations of the American elm have been devastated by pathogenic fungi of the genus *Ophiostoma,* the causal agent of Dutch elm disease (DED). Populations of *Ophiostoma* have been separated on the basis of aggressiveness and phenotype characteristics resulting in the establishment of three distinct species, the less aggressive *O. ulmi* (formerly known as *Ceratocystis ulmi*), the highly aggressive *O. novo-ulmi*[1] and *O. himal-ulmi*, a species endemic to the western Himalayas [2]. During the last century there have been two destructive epidemics of the disease in Europe and North America caused by successive introductions of this pathogen. The less aggressive *O. ulmi* was first introduced to Western Europe in 1918 and then arrived in America on imported timber in 1928. This first disease wave was relatively benign, and killed only a small proportion of elms, more often simply causing dieback in select branches. The disease had largely dissipated by 1940 possibly due to its susceptibility to viruses [3]. The second, more aggressive wave of the disease, caused by *O. novo-ulmi*, was first reported in the United States in 1930 [3]. In Canada *O. novo-ulmi* was first observed in Quebec in 1944, and then progressed eastwards reaching the Atlantic coast in 1969. It is presently moving westward from the epicentre threatening elm populations in Saskatchewan and Alberta. The city of Winnipeg, which has the largest urban elm population in Canada, has lost 21,606 trees during the last four years. The city’s elm population now numbers 140,000 and the city continues to lose between 4000 to 5600 elm trees each year and spends approximately $2.7 million per year, plus another contribution of $1.0 million by the province, on sanitation and pruning [4]. There are currently no effective methods to control the spread of DED. Traditionally the focus has been on fungicides to stem the growth of the fungus or pesticides to control the spread of the insect vector. Treatment with pesticides proved to be a very expensive option and was not very effective as the beetles simply moved to other tree species during fumigation [5] while fungicide treatments were deemed to be too expensive and not very effective [6]. An attractive alternative to the use of chemical pesticides or fungicides is the development of a biological control for *O. novo-ulmi*. This requires, at minimum, an agent which is antagonistic to the fungus, is transmissible to extant populations of the fungus in the field, and is very specific to minimize off-target effects. One such agent might be found within the mycoviruses which have been reported in all classes of fungi. In many cases, these viral infections do not cause disease symptoms in their hosts however some mycoviruses reduce the ability of their hosts to cause disease in plants [7]. This property, known as hypovirulence, could provide a measure of biological control [8,9]. All hypovirulence-associated mycoviruses described to date have double-stranded (ds) or single-stranded (ss) RNA genomes and include representatives of the *Totiviridae*, *Chrysoviridae*, *Narnaviridae,* and *Reoviridae.*

Mycoviruses are usually located in the cytoplasm of the fungal host however certain double-stranded RNA (dsRNA) viruses are found exclusively in the mitochondria [9,10]. This latter class, referred to as mitoviruses, have no capsid and encode an RNA-dependent RNA polymerase (RdRp) that is required to replicate the RNA [11,12]. There are twenty-five fully characterized species of genus *Mitovirus* listed in the National Center for Biotechnology Information (NCBI) Genome database, seven of which are found in the fungal genus *Ophiostoma*.

Population genetic studies of the pathogen at the western Canadian disease front demonstrated that there was little diversity in the *O. novo-ulmi* isolates surveyed. Over a nine-year period we observed no increase in the diversity of vegetative compatibility (*vc)* types or nuclear genotypes and populations of the pathogen in western Canada are essentially represented by two very large clones [13]. This scenario is very different in Europe where the pathogen has very quickly established a variety of *vc* types behind the disease front, typically within a period of six to ten years [14]. It was hypothesized that one of the major drivers for this diversification was the presence of deleterious mitoviruses [15]. It would therefore be anticipated that the clonal populations in western Canada would be relatively free of dsRNA viruses. In both the 1993 and 2002 sample set, we were only able to find one isolate in each group infected with dsRNA [13]. Both isolates were found in close proximity and their dsRNA profiles were identical in size and banding pattern, suggesting spread of this dsRNA has been limited. These dsRNAs were resistant to DNase and S1 nuclease while susceptible to degradation by RNase and could be transferred to naïve isolates of *O. novo-ulmi* by hyphal anastomosis [13]. It is curious that these dsRNAs were found infecting an individual member of the large clonal population raising the question of their origin.

We describe here the sequence characterization of two RdRp encoding mitoviruses as well as two ancillary dsRNA molecules lacking coding function from *O. novo-ulmi* isolate 93–1224 . We have named the RdRp encoding viruses OnuMV1c [GenBank*:* KF026355] and OnuMV7 [GenBank*:* KF031943] based on the classification of other *Ophiostoma* mitoviruses characterized in Europe*.*

## Results

### cDNA synthesis and sequence analysis

**Figure 1:**
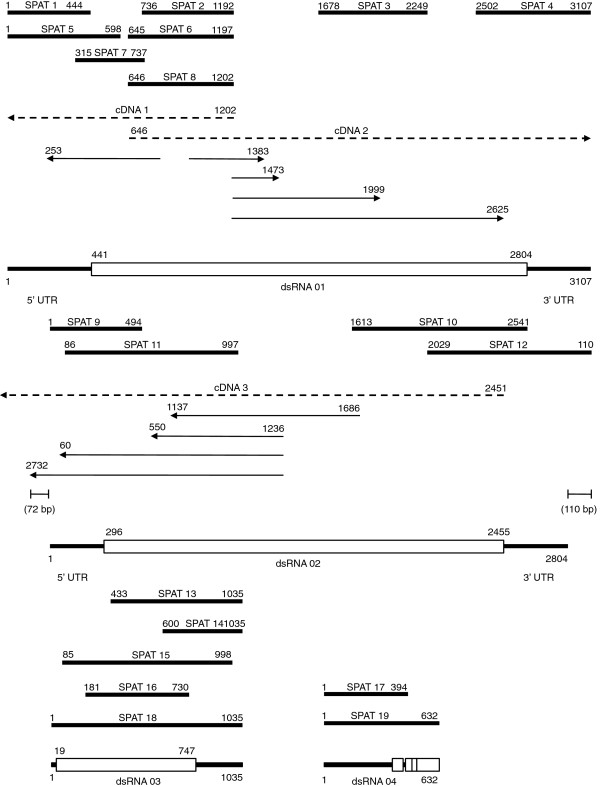
**Schematic representation of alignment of a series of SPAT and partial cDNA clones derived from the dsRNAs of *O. novo-ulmi *isolate 93–1224.** Contiguous sequences were initially collated by alignment of SPAT clones (black bars). The primers 5^′^TGCAATTTGTTGCTAGTGGA3^′^ and 5^′^ACCTGCAACAAGTAACAATCTGG3^′^ were used to make cDNA 1 and cDNA 2 according to SPAT 8 and the primer 5^′^CTATATACAGTTAATATTAATTACAGGTAGATATGCTATGATATTTACAAATATCACTTATTAAACG3^′^ was used to make cDNA 3 according to SPAT 10 (dashed lines). The linkages between contigs were determined by chromosome walking (indicated by lines with arrows) leading to a final assembly for dsRNAs 01–04. A single large ORF (white boxes) with the potential to encode RNA-dependent RNA Polymerases (RdRPs) was predicted for dsRNAs 01 and 02 while other smaller ORFs were detected in dsRNAs 03 and 04.

The application of the single primer amplification technique (SPAT) to purified dsRNA from *O. novo-ulmi* 93–1224 as a template yielded nineteen unique cDNA clones many of which showed sequence similarity to RdRps (Figure 1). The sequences were compared to the genomic sequences of *O. novo-ulmi* H327 to determine whether there was any sequence similarity to known nuclear or mitochondrial sequences [16]. Each of the clones were unique to isolate 93–1224. Where possible overlapping SPAT clones were assembled into continuous sequences. Many of the SPAT clones ended at the same position suggesting the ends of discrete dsRNA molecules. To facilitate linkage between SPAT clones, cDNAs were constructed according to characterized sequence of SPAT clones 8 and 10 and the gap regions determined by chromosome walking. Four separate complete contigs were developed corresponding to dsRNA 01 (3107 nt), dsRNA 02 (2804 nt), dsRNA 03 (1035 nt) and dsRNA 04 (632 nt) (Figure 1). None of the sequences were polyadenylated. 5′ Rapid Amplification of cDNA Ends (RACE) confirmed that dsRNA 01 was a linear molecule with the ends defined by SPAT 1 and SPAT 4. The last 110 bp of SPAT 12 overlapped with the first 110 bp of SPAT 9 suggesting that dsRNA 02 was either a closed circular molecule or occurred as a series of concatemers (Figure 1). This was confirmed by chromosome walking from SPAT 12 which extended the 5′ untranslated region (UTR) sequence a further 72 bp into the next repeating unit and discrete ends to the repeating unit could not be determined (Figure 1). The nominative 5′ and 3′ UTRs for dsRNA 02 were assigned according the 5′ end of SPAT 9 as five independently derived versions of this clone ending at this position were discovered. The two smallest contigs, representing dsRNA 03 and dsRNA 04 appeared to be linear. There was no sequence similarity between any of the four contigs.

### Coding potential

**Figure 2:**
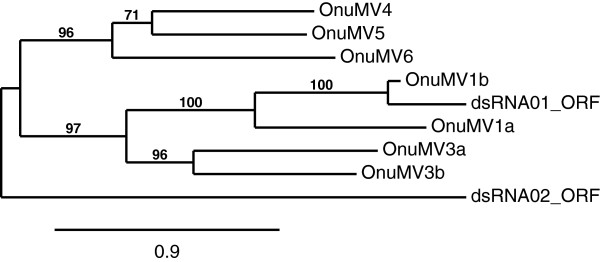
**Unrooted maximum likelihood tree for the ORFs of dsRNA 01 and dsRNA 02 from *O. novo-ulmi *isolate 93–1224 with all RNA-dependent RNA polymerases (RdRPs) encoded by *Ophiostoma *mitoviruses.** The dsRNA 01 clustered closely with *O. novo-ulmi* mitovirus OnuMV1b through was not con-specific. The dsRNA 02 did not cluster with any other *Ophiostoma* mitovirus ORF and was an outlier for this group. Virus notations are as follows: OnuMV1a [GenBank*:* CAJ32466.1] = *O. novo-ulmi* mitovirus 1a; OnuMV1b [GenBank*:* CAJ32467.1] = *O. novo-ulmi* mitovirus 1b; OnuMV3a [GenBank*:* CAA06228.1] = *O. novo-ulmi* mitovirus 3a; OnuMV3b [GenBank*:* CAJ32468.1] = *O. novo-ulmi* mitovirus 3b; OnuMV4 [GenBank*:* CAB42652.1] = *O. novo-ulmi* mitovirus 4; OnuMV5 [*NCBI Reference Sequence:* NP_660180.1] = *O. novo-ulmi* mitovirus 5; OnuMV6 [*NCBI Reference Sequence:* NP_660181.1] = *O. novo-ulmi* mitovirus 6.

**Figure 3:**
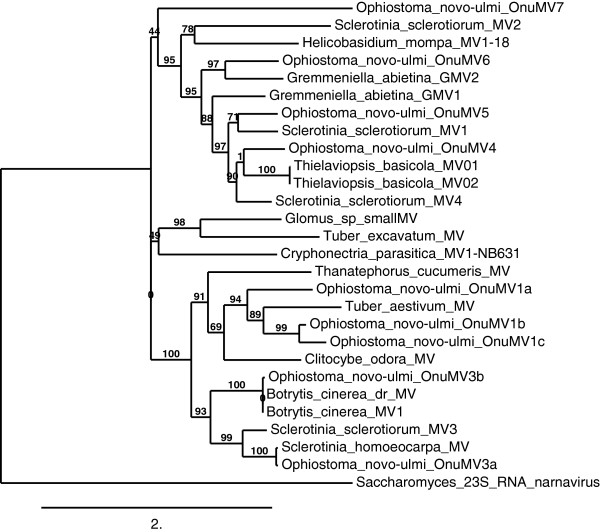
**Phylogenetic identification of OnuMV1c and OnuMV7.** The fungal mitovirus RdRp sequences were obtained from the NCBI gene bank database. Multiple sequence alignments were performed using MUSCLE and the tree constructed using NJ method with 1000 bootstrap replicates. Bootstrap values are shown. The WAG substitution model was selected assuming an estimated proportion of invariant sites (of 0.025) and 4 gamma-distributed rate categories to account for rate heterogeneity across sites. *Saccharomyces* 23S RNA narnavirus [*UniProt:* Q07048] = RdRp *Saccharomyces* 23 S RNA narnavirus PE = 1 SV = 2 served as an outgroup. Virus notations were according to Figure 2 and as follows: *Sclerotinia sclerotiorum* MV2 [GenBank*:* AEX91879.1] = *S. sclerotiorum* mitovirus 2; *Helicobasidium mompa* MV1-18 [GenBank*:* BAD72871.1] = *H. mompa* mitovirus 1 – 18; *Gremmeniella abietina* GMV2 [GenBank*:* AEY76153.1] = *G. abietina* non-host-specific mitrochondrial RNA virus S1; *Gremmeniella abietina* GMV1 [GenBank*:* CCD32685.2] = RdRp *Gremmeniella* mitovirus; *Sclerotinia sclerotiorum* MV1 [GenBank*:* AEX91878.1] = *S. sclerotiorum* mitovirus 1; *Thielaviopsis basicola* MV01 [*NCBI Reference Sequence:* YP_002822229.1] = RdRp *T. basicola* mitovirus; *Thielavopsis basicola* MV02 [GenBank*:* AAZ99833.1] = RdRp *T. basicola* mitovirus; *Sclerotinia sclerotiorum* MV4 [GenBank*:* AGC24233.1] = RdRp *S. sclerotiorum* mitovirus 4; *Glomus sp.* Small MV [GenBank*:* BAJ23143.1] = Putative RdRp *Glomus sp.* RF1 small virus; *Tuber excavatum* MV [GenBank*:* AEP83726.1] = RdRp *T. excavatum* mitovirus; *Cryphonectria parasitica* MV1-NB631 [*NCBI Reference Sequence:* NP_660174.1] = RdRp *C. parasitica* mitovirus 1-NB631; *Thanatephorus cucumeris* MV [GenBank*:* AAD17381.1] = dsRNA viral RdRp *T. cucumeris*; *Tuber aestivum* MV [*NCBI Reference Sequence:* YP_004564622.1] = RdRp *T. aestivum* mitovirus; *Clitocybe odora* MV [*NCBI Reference Sequence:* YP_005352912.1] = RdRp *C. odora* virus; *Botrytis cinerea* dr MV [*NCBI Reference Sequence:* YP_002284334.1] = RdRp *B. cinerea* debilitation-related virus; *Botrytis cinerea* MV1 [GenBank*:* ABQ65153.3] = RdRp *B. cinerea* mitovirus 1; *Sclerotinia sclerotiorum* MV3 [GenBank*:* AGC24232.1] = RdRp *S. sclerotiorum* mitovirus 3; *Sclerotinia homoeocarpa* MV [GenBank*:* AAO21337.1] = *S. homoeocarpa* mitovirus.

The nucleotide sequence of the dsRNA contigs were examined for the presence of open reading frames (ORFs) in all six reading frames. When the universal codon usage for cytoplasmically translated proteins was applied, there were no long ORFs however shorter segments of RdRp-like genes could be recognized. Because of the high similarity of these RdRp fragments to mitoviruses, a mitochondrial-specific codon usage pattern was applied. When the genetic code for mold, protozoan, coelenterate mitochondrial and mycoplasma (code 4) was employed, a single large ORF was found on the positive strand of both dsRNA 01 and dsRNA 02 (Figure 1). The ORF of dsRNA 01, which started with an AUG - start codon, and terminated with a UAG - stop codon, had the potential to encode a protein of 788 amino acids. Similarly a single large ORF was found on the positive strand of dsRNA 02, having an AUG - start codon and a TAA - stop codon, had the potential to encode a protein of 720 amino acids. According to Basic Local Alignment Search Tool (BLAST) analysis the dsRNA 01 ORF had a very high sequence similarity to the *Ophiostoma* RdRp encoded by the mitovirus OnuMV1b having a 70% of maximum amino acid identity for 97% of the query coverage. There was a significantly higher degree of sequence identity in the C-terminal region as compared to the N-terminal region. Alignment of the first 261 amino acids of OnuMV1c to the first 250 amino acids of OnuMV1b revealed only a 28% percent sequence identity while there was 88% sequence identity in the remaining 527 amino acid residues compared to a similar 526 amino acid region of OnuMV1b. Less significant but obvious identity existed in amino acid sequences with other mitoviruses clearly demonstrating a close relationship between this newly described dsRNA and other mitoviruses. Following a BLAST search for the ORF of dsRNA 02 it was found to be most similar to the RdRp of *Gremmeniella* mitovirus with a maximum identity of 30% for a 50% query cover. This molecule had only had a 29% identity for 49% query cover of *Ophiostoma* mitovirus OnuMV3a RdRp and a 35% identity for a 30% query cover of *Ophstiostoma* mitovirus OnuMV4, Construction of a phylogeny of all the mitovirus RdRp sequences for *Ophiostoma*, including those encoded by dsRNAs 01 and 02, demonstrated a close clustering of the dsRNA01 ORF with OnuMV1a and OnuMV1b hence we named this new mitovirus OnuMV1c (Figure 2). The RdRp encoded by the dsRNA 02 appeared to be unique and did not cluster with any other mitovirus previously described for *O. novo-ulmi* hence we named this second new mitovirus OnuMV7 following the numbering convention of dsRNAs that encode distinct RdRp-like proteins as proposed by Hong [17] and Doherty [11] (Figure 2). Phylogenetic comparison of the newly described OnuMV1c RdRp gene to a larger group of all characterized fungal mitoviruses indicated that this virus again grouped in a distinct clade containing *Ophiostoma* mitoviruses OnuMV1a, 1b, and also included OnuMV3a and 3b (Figure 3). Interestingly the RdRp of the *Tuber aestivum* MV clustered tightly with the OnuMV1a, 1b, 1c group. The RdRps of *Sclerotinia sclerotiorum* MV3 and *Sclerotinia homoeocarpa* MV clustered with OnuMV3a while those of *Botrytis cinerea* MV1 and *Botrytis cinerea* dr MV clustered tightly with OnuMV3b. While the newly described OnuMV7 did group in a clade containing OnuMV 4, 5, and 6 there was no close association to any of these *Ophiostoma* viral species nor with any other fungal virus species. The 729 bp ORF of dsRNA 03 had the potential to encode a polypeptide of 243 amino acids however there was no similarity of this ORF to any RdRp or to any other viral protein (Figure 1). There were four very small ORFs found on dsRNA 04 which shared limited sequence similarity to the 5′ ends of several mitovirus RdRps and had the highest similarity to the 5′ region of OnuMV1b. The RdRp homologous region of dsRNA 04 was, however, incomplete and interspersed with stop codons hence was unlikely to encode a functional enzyme (Figure 1). Both dsRNA 03 and dsRNA 04 are considered to be defective RNAs, the replication of which likely depends on a functional RdRp from some other source. Unlike dsRNAs 01 and 02, these molecules were not observed by gel electrophoresis and were much less abundant.

### Sequence similarities between *Ophiostoma* mitoviruses

**Figure 4:**
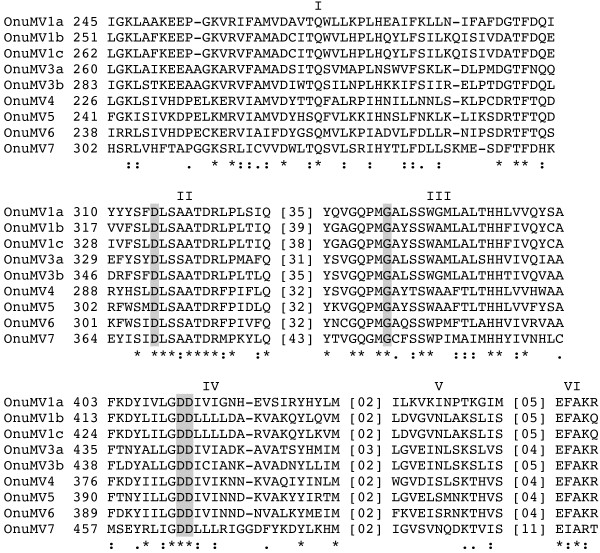
**Alignment of the RdRp conserved amino acid motifs of *O. novo-ulmi *encoded by mitochondrial viruses.** Virus notations and labelling of the motifs follow Hong et al. [18]. Symbols shown below the alignment indicate identical amino acids (*), as well as higher (:) and lower (.) chemically-similar residues, respectively, as defined in the CLUSTAL W program. Analysis of the amino acid sequence of OnuMV1c showed at least three common motifs (shaded) that are typically conserved in the sequences of all polymerases showing RNA template specificity. Numbers in parentheses represent the number of amino acid residues between motifs.

Alignment of the RdRp encoded by OnuMV1c and 7 with accordant regions of all other *Ophiostoma* mitoviruses (OnuMV1a, 1b, 3a, 3b, 4, 5, and 6) revealed three well-conserved motifs (labeled as Motifs II, III and IV in Figure 4) and three less conserved motifs [17] (labeled as Motifs I, V and VI). Also recognized were conserved amino acids D in Motif II, G in Motif III, DD in Motif IV that are common to all other RNA virus genomes [18]. Within Motif I of the *Ophiostoma* mitoviruses there were seven positions showing identical amino acids and an additional fifteen positions having chemically similar amino acids. Core conserved features of Motif II consisted of DLS-A/S-ATDR-F/L/M-P. Motif III consisted of GQ-P/G-MG-AC-Y/L/Q/F-S/T-SW and each of Motifs IV and VI consisted of L/I-GDD and E-F/I-AK/R respectively (Figure 4). For this group of mitoviruses most of the variability in these motifs was found in OnuMV7 which was the least conserved mitovirus species overall. Part of conserved Motif IV (GDD) also corresponded to the conserved region VI in an alignment of viruses of the family *Partitiviridae*[19].

### Northern detection of ssRNA and dsRNA

**Figure 5:**
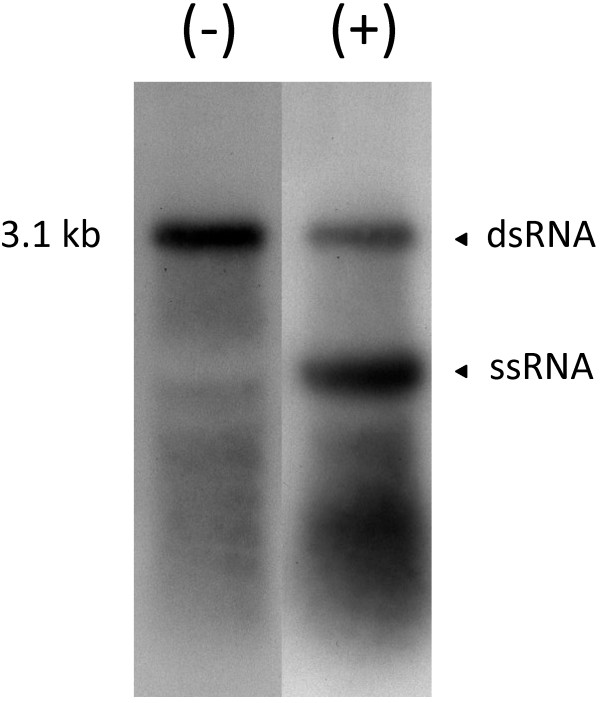
**Northern blot hybridization using strand-specific probes of *O. novo-ulmi *93–1224 mitovirus OnuMV1c.** Total RNAs were extracted and probed with either (−) or (+) strand specific probes corresponding to positions 35 to 910 of the RdRP coding region of the OnuMV1c. Double stranded RNA (dsRNA) was detected with both probes and single stranded transcripts (ssRNA) was detected only when (+) stranded probes was used. The ssRNA ran further down the gel.

Northern hybridization analysis revealed that when total RNA was hybridized with (−) strand-specific probe derived from OnuMV1c there was a major discrete signal at 3.1 kbp, corresponding to the size of the dsRNA 01 observed by gel electrophoresis. When the blot was probed with the (+) strand-specific probe the same 3.1 kbp dsRNA signal was observed as well as a second signal migrating further down the gel corresponding to the single-stranded transcript (ssRNA). As estimated from the intensity of the hybdridization signal the single stranded transcript was in greater abundance than the dsRNA (Figure 5). A similar pattern was observed when the blots were probed with OnuMV7 probes except that the probes hybridized to a 2.8 kbp dsRNA (not shown).

### Potential secondary structures

**Figure 6:**
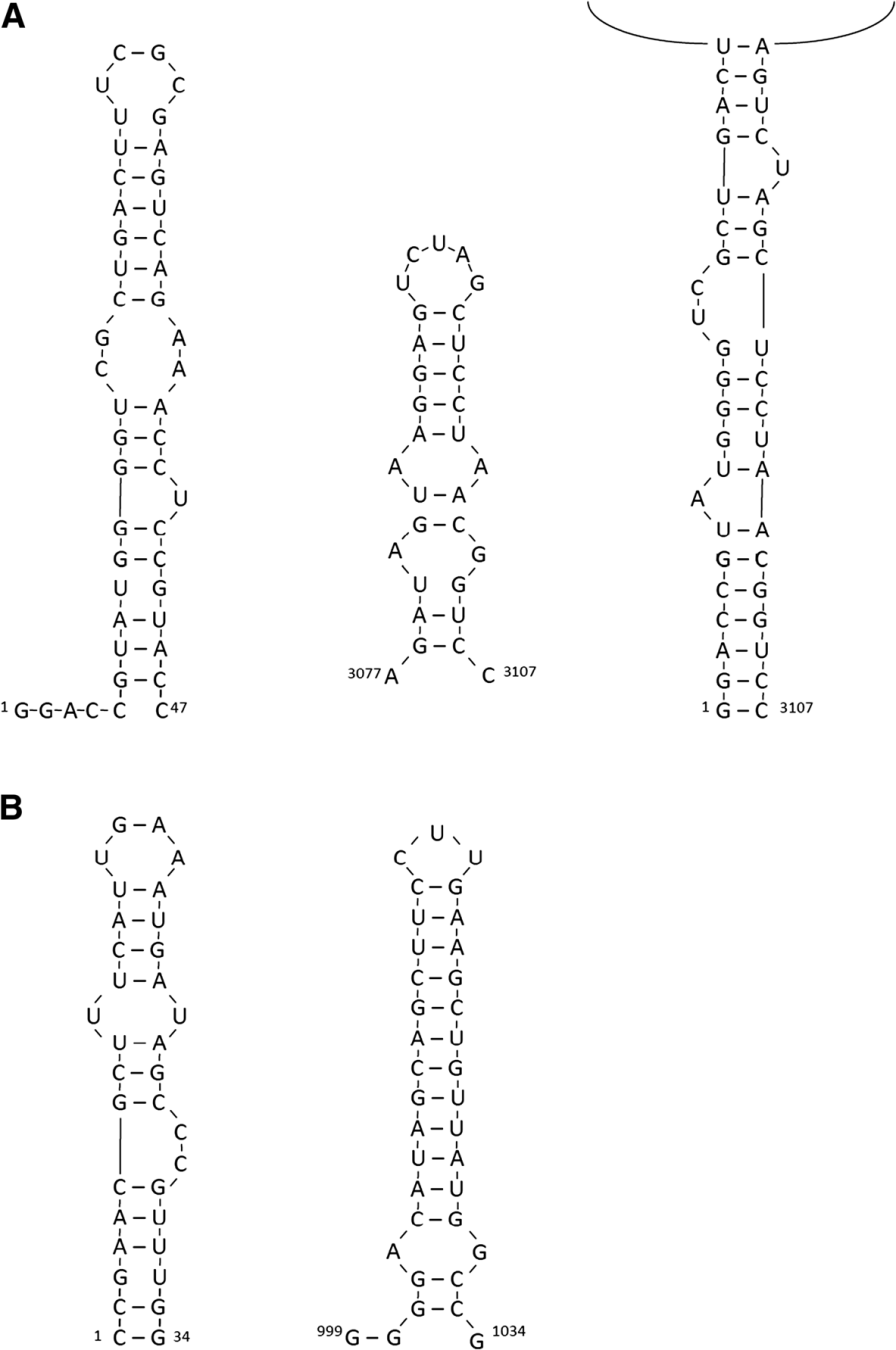
**Potential secondary structures of the ends of dsRNA01 (OnuMV1c) and dsRNA03. A**. The potential 5^′^ and 3^′^ terminal snap-back secondary structures of *O. novo-ulmi* mitovirus OnuMV1c in isolate 93–1224 are shown along with a potential panhandle structure of the (+) strand. **B**. Potential snap-back structures were located at the 5^′^ and 3^′^ ends of the dsRNA 03 which lacked significant coding function. An energetically favorable panhandle structure could not be definitively located for dsRNA03.

The 5′- and 3′- UTRs of dsRNA 01 (OnuMV1c), measuring 440 and 303 bp in length respectively, were examined for potential secondary structures using the RNAfold algorithm which predicts the structure summarizing free positive or negative energy change associated with all possible pairing. An examination of the positive strand of the RNA sequence showed that the first 47 bp of 5′- terminal sequence of the positive strand (^1^GGACCGUAUGGGGUCGCUGACUUUCGCGAGUCAGAAACCUCCGUACG^47^) could potentially be folded into a double-stranded stem-loop structure (free energy −24.11 kcal/mol) with 4 unpaired nucleotides at the 5′ end (Figure 6A). The 30 bp of 3′- terminal sequence (^3077^AGAUAGUAAGGAGUCUAGCUCCUAACGGUCC^3107^) also had the potential to be folded into a double-stranded stem-loop structure with free energy −11.25 kcal/mol (Figure 6A). A potential panhandle structure between the 5′ and 3′ UTR regions was also predicted with a free energy of −20.56 kcal/mol (Figure 6A). There were no obvious stem-loop structures or panhandle structures in the upstream or downstream UTRs of dsRNA 02 (OnuMV7). This was consistent with the finding that this molecule occurred as either a closed circular molecule or occurred as a concatemer. The dsRNA 03 had stem-loop structures at both ends of the molecule corresponding to (^1^CCGAACGCUUUCAUUGAAAUGAUAGCCCGUUUGG^34^) with a free energy of −10.88 kcal/mol and (^999^GGGGACAUAGCAGCUUCCUUGAAGCUGUUAUGGCCG^1034^) with a free energy of −19.67 kcal/mol (Figure 6A). While there was potential to form a pan-handle structure the likelihood of snap back to the stem-loop structure was much greater. The dsRNA 04 had a stem-loop structure at the 5′ terminus of the molecule but not at the 3′ terminus and may in fact represent an incomplete or truncated sequence (not shown).

## Discussion

### Mitovirus diversity

Fungal viruses or mycoviruses are widespread among fungi. Although the first definitive description of mycoviroses was published just over 50 years ago [20], they have been found to be quite common and are usually associated with double-stranded ribonucleic acid (dsRNA) elements. Some of the fungi known to harbor dsRNA viruses do not appear to display any associated phenotype while the impact on others can result in severe debilitation [21-23]. Certain infections can result in reduced virulence or pathogenicity of the fungal pathogen within their host organism [24]. A defining feature of mitoviruses is that they inhabit mitochondria and utilize the mitochondrial codon preference as opposed to the “universal” codon preference of the cytoplasm. Many mitochondrial viruses have a single ORF that encodes a unique RdRp. Studies on the diversity of viruses of European isolates of *O. novo-ulmi* demonstrated that there were a variety of novel mitoviruses within populations of *O. novo-ulmi* at the disease fronts. A total of thirteen dsRNAs were isolated from several isolates [25] and one diseased isolate, *O. novo-ulmi* Log1/3-8d2 (Ld), was reported to be multiply infected with twelve distinct mitoviruses [26]. The complete genome sequences have been determined for *Ophiostoma* dsRNAs corresponding to OnuMV3a, OnuMV4-Ld, OnuMV5-Ld, OnuMV6-Ld [17]. RNA-7 and RNA-10, which did not encode an RdRp, were derived from OnuMV4-Ld which serves as a helper virus for the replication of these two molecules [17]. The RdRp sequences of *O. novo-ulmi* mitoviruses OnuMV1a, OnuMV1b and OnuMV3b were subsequently determined [11]. Mitoviruses are relatively rare at the western Canadian disease front having been documented on only two occasions in the City of Winnipeg. Infected *O. novo-ulmi* isolates 93–1224 and 02–0833 were collected from the same region of the city in 1993 and 2002 respectively and the dsRNAs were identical in size and banding pattern [13]. We have determined the complete genome sequence of two mitoviruses isolated from *O. novo-ulmi* (isolate 93–1224) and propose that the viruses OnuMV1c and OnuMV7 are two new members of the genus *Mitovirus* in the family Narnaviridae. This is supported by their utilization of the mitochondrial codon usage pattern, the presence of an ORF with a potential to encode an RdRp on the positive strand, and the presence of a double stranded stage in their replicative cycle. The most compelling evidence for the classification of dsRNA01 as OnuMV1c was the high similarity of its encoded RdRp to the RdRp encoded by mitovirus OnuMV1b. The high degree of conservation of the C-terminal regions of OnuMV1c and OnuMV1b and the very low conservation of the N-terminal regions could suggest that OnuMV1c may have been recently derived from OnuMV1b through a recombination event with another mitovirus species leading to an exchange of the N-terminal regions. Alternately the approximately 250 amino acid N-terminal regions of mitoviruses may be under lower selective pressure leading to an accelerated genetic divergence for this region. Motifs typical of RdRps were found exclusively in the C-terminal region past amino acid positions 251 for OnuMV1b and 262 for OnuMV1c. The second complete mitovirus found in 93–1224, corresponding to dsRNA02, was named OnuMV7 as it was very distinct from any other mitovirus yet described for *Ophiostoma*. While the key elements of the RdRp motifs could be recognized in the OnuMV7 RdRp, this mitovirus did not seem to be recently derived from any other *Ophiostoma* mitovirus. For isolates infected with multiple mitoviruses it is not known whether each unique RdRp is exclusively responsible for the replication of its own mitovirus or whether each RdRp might associate with multiple mitoviruses, or indeed other dsRNAs in the same host. The presence and maintenance of complete reading frames for both OnuMV1c and OnuMV7 would argue for two functional RdRps each with their own specificity. We also found two additional dsRNAs (dsRNA03 and dsRNA04) which did not encode functional RdRp and represent defective or degenerated RNAs. As was seen for the defective dsRNAs (RNA 7 and RNA 10) in Europe, dsRNA04 contained a degenerated RdRp quite similar to that of the OnuMV1c cluster of viruses while dsRNA03 did not have the potential to code for an RdRp. It appears that the two additional dsRNAs, dsRNA03 and dsRNA04, rely on the RdRp encoded by one or both of OnuMV1c or OnuMV7, for their replication. This question can only be resolved by the development of new strains of *O. novo-ulmi* isogenic for each of the mitoviruses. In this way the functionality of each RdRp can be assessed.

### Viral replication

Mitovirus RdRp associates with its own RNA to form an RNA/RdRp complex that plays a key role in RNA replication in mitochondria of the host [27]. It has been suggested that terminal residues at the 5’ and 3′ UTRs of linear mitoviruses act as stem-loop structures for RdRp recognition and initiation of replication [24]. It has also been suggested that the potential for RNA to be folded into a secondary pan-handle structure at the 5′ and 3′ ends may be a diagnostic feature of mitoviruses [17] and that these structures may act as promoters for RNA replication. Such structures were predicted in *Chalara elegans* mitovirus (CeMV) [28], OnuMV3a [17], and *Sclerotinia homoeocarpa* mitovirus [23]. Terminal stem-loop structures that could also be folded as a pan-handle structure were predicted for OnuMV1c however OnuMV7 apparently lacked these structures. The mechanism for replication of OnuMV7 might be quite different from other mitoviruses described to date and may involve a rolling circle mechanism as suggested by the resolution of the genome sequence as either a circular molecule or a series of concatemers. The termini of RNA03 formed stem-loop structures typical of mitoviruses yet did not encode an RdRp. There was no homology between the ends of any of these elements hence recognition by the RdRp is likely not strictly sequence based and might be structure based. This raises the potential for engineering these apparently defective dsRNAs to include other genetic elements, such as anti-sense or interfering RNAs, and co-infecting naïve hosts with both the engineered molecule and a helper mitovirus encoding an RdRp that would thus provide replication capacity for both.

### Origin of the Canadian mitoviruses

The rarity of OnuMV1c at the western Canadian disease front raises the question of the origin of this virus. These viruses are transmitted intracellularly: vertically during host cell division and sporogenesis and horizontally during cell fusion as a result of hyphal anastomosis. Horizontal transmission usually occurs only between individuals of the same species or closely related *vc* groups [29]. It is clear that isolate 93–1224, being a member of the dominant clone currently found at the disease front, acquired the virus infection after its arrival in western Canada, most likely through a transient hyphal anastomosis. Because there were two waves of infection spread through Europe and North America with the less aggressive *O. ulmi* being replaced by the more aggressive *O. novo-ulmi* it may be that the virus was harboured in the older and ancestral infection wave. Usually, when *O. novo-ulmi* arrives at a “new” area it rapidly replaces resident *O. ulmi*[7]. During this replacement process, the close proximity of *O. ulmi* and *O. novo-ulmi* in the bark beetle galleries provides the physical opportunity for interspecific genetic exchange. Sexual hybridization between these two *Ophiostoma* species is quite rare but there may be an opportunity for virus transfer through transient hyphal anastomosis [30]. A preliminary comparison of viruses in *O. ulmi* and *O. novo-ulmi* isolates obtained from the same epidemic front site in Europe indicated a very close similarity in their RNA sequences. *Ophiostoma novo-ulmi* 93–1224 may thus have become infected with debilitating virus infections from *O. ulmi*[31].

There is also a possibility of horizontal transfer from other yet unidentified species. Interestingly mitovirus OnuMV3a-Ld is con-specific with a hypovirulence associated dsRNA from *S. homoeocarpa*[23] and mitovirus OnuMV3b is con-specific with a hypovirulent virus found in *B. cinerea*[32] suggesting that horizontal transmission between these different fungal groups may have occurred [23]. Further screening of *O. novo-ulmi* isolates and other fungi for mitoviruses in the city of Winnipeg could better address the question of the origin of OnuMV1c and OnuMV7. Studies of fungal viruses and hypovirulence can increase our understanding of molecular mechanisms influencing the expression of virulence in these plant pathogens and broaden the potential of fungal viruses as a biological control.

## Conclusions

The greatest interest in studying mitoviruses lies in the potential to use them as a biological control of pathogenic fungi. Mitovirus infection can affect physiological and biochemical processes and even change the morphology of fungi [9,33,34]. Some mitoviruses infections can be latent, whereby the virus is present but does not cause disease symptoms. This could benefit the fungal host by conferring protection against infection by other viruses [12] as is the case for OnuMV3a in *S. homoeocarpa*[23], *Rhizoctonia solani*[34] and CeMV in *C. elegans*[28]. The use of virus-induced hypovirulence as a biological control relies on the ability to transfer the virus between isolates within a population of the target pathogen. RNA viruses that have been found in *O. novo-ulmi* to date are located in mitochondria and can only be transmitted during anastomosis between compatible hyphae, or induced forms of cytoplasmic mixing. The efficiency of dissemination of hypoviruses is inversely related to the *vc* diversity of their hosts. The disease front in western Canada, being essentially composed of two very large clones, provides an ideal target for deployment of such a biological control.

## Methods

### Fungal growth and culture maintenance

Stock cultures of *O. novo-ulmi* 93–1224 (collected by P. Pines from an infected elm in Winnipeg in 1993) were stored frozen at -70°C in 10% v/v glycerol. Cultures were grown on solid *Ophiostoma* complete media (OCM) [35] at 23°C and kept at 4°C for short-term storage. In preparation for dsRNA purification *O. novo-ulmi* mycelium was grown in liquid OCM medium, harvested by centrifugation at 2000 g for 10 min, flash frozen in liquid nitrogen and crushed to a fine powder using a chilled mortar and pestle. The dsRNA was extracted [13] and visualized by staining with GelRed stain (Biotium Inc., Burlington, ON.) after electrophoresis on 1.0% agarose in 1× TAE buffer (0.04 M Tris-acetate; 1 mM EDTA) at 100 V for 60 min.

### SPAT and full length amplification of cDNA (FLAC)

The SPAT approach was used to synthesize cDNA from a dsRNA template [36]. The N-blocked primer PC3 (5′-PO_4_-AGGTCTCGTAGACCGTGCACC -NH_2_-3′) was ligated to the 3′ end of gel-purified dsRNAs. Approximately 250 ng of PC3 primer was ligated to 200 ng of purified dsRNA at a molar ratio of approximately 40:1. The ligation mixture included: 50 mM HEPES/NaOH, pH 8.0 (Fermentas), 20 mM MgCl_2_, 0.01% BSA (Promega, Mannheim, Germany), 1 mM ATP (Fermentas), 3 mM DTT (Roche, Mannheim, Germany), 10% (v/v) DMSO (Sigma–Aldrich), 20% (w/v) PEG8000 (Fermentas), 20 units of RNaseOUT™ RNase inhibitor (Invitrogen), and 30 units of T4 RNA ligase (Fermentas) in a final volume of 30 μL. The ligation components were incubated for 6 h at 37°C, 1 h at 18°C, followed by overnight incubation at 12°C. The primer-ligated dsRNAs were purified from excess primer using a NucleoSpin® Extract II column and concentrated in a SpeedVac vacuum concentrator for 15 min. The purified primer-ligated dsRNAs were denatured at 98°C for 2 min in the presence of 1.0 M Betaine and 2.5% (v/v) DMSO followed by quenching on ice for 5 min. The cDNA synthesis reaction mixture contained: 50 mM Tris–HCl (pH 8.3 at 25°C), 75 mM KCl, 3 mM MgCl_2_, 10 mM DTT, 1 mM dNTPs, 20 units of RNaseOUT™ inhibitor, and 400 units of Maxima Reverse Transcriptase (Fermentas). The reaction mixture was incubated for 1 h at 50°C followed by 15 min at 55°C. The RNA-cDNA mixture was digested with 0.1 M NaOH for 20 min at 70°C and was neutralized by the addition of 0.1 M Tris–HCl pH 7.5, 0.1 M HCl. The amplification mixture, adjusted to a final volume of 25 μL, contained 5 μL of cDNA, 320 μM of each dNTP, 2 mM MgCl_2_ and 1.25 μM of primer (5′GCACGGTCTACGAGACCT-3′) and 2.5 units of Go Taq DNA polymerase (Promega) plus corresponding 1 X buffer: The mixtures were incubated in a Biometra T professional Thermocycler for 2 min at 72°C followed by 2 min at 95°C and then subjected to 40 cycles of denaturation at 95°C for 25 s with an increment of 1 s per cycle, annealing at 65°C for 30 s and extension at 68 C for 5 min. This was followed by a final extension at 72°C for 10 min. Amplification products were cloned into pGEM-T vector and transformed into *E. coli* competent cells. The sequences were determined using Sanger sequencing with an ABI 3730XL sequencer (Eurofins MWG Operon, Ebersberg, Germany). Cloned cDNAs, putatively corresponding to dsRNA sequences, were assembled into overlapping contiguous sequences (contigs). The cloned cDNAs were also screened for similarity to characterized mitovirus sequences from the GenBank database using the Basic Local Alignment Search Tool (BLAST) [37].

### Gene walking

Following the initial identification of RdRp-like gene fragments from the dsRNA preparations the full-length sequences of the target viruses were determined by primer extension and gene walking. cDNAs, corresponding to select cloned dsRNA fragments, were synthesized using the protocol for first-strand cDNA synthesis from Omniscript Reverse Transcription Kit (Qiagen, Toronto, ON). These target cDNAs were then amplified using a combination of genome specific primers paired with random sequence primers (9mers) or from primer designed according to other SPAT clones to extend the characterized sequence. The terminal sequences of the largest linear dsRNA molecule were determined using the 5′RACE Kit (Invitrogen, Grand Island, NY). This was possible because even though mitoviruses are predominantly regarded as having a single stranded RNA genome, there is a double stranded RNA stage in their replication cycle [10,38] which permitted the use of 5′RACE to determine the sequence of both the positive and the negative strand of the dsRNA. Potential coding regions were detected using ORF Finder [39]. RNA secondary structures were determined using the program RNAfold [40]*.*

### Northern blot hybridization with strand-specific probes to the dsRNAs

In preparation for northern analysis dsRNAs from strain 93–1224 were separated by non-denaturing electrophoresis on a 1% agarose gel which was then soaked in NaOH (50 mmol/L) and NaCl (0.15 mol/L) for 15 min, followed by soaking in 10X SSC (1X SSC is NaCl, 0.15 mol/L, plus sodium citrate, 0.015 mol\L) for 10 min before being transferred to a nylon membrane GeneScreen Plus® (NEM™. Prehybridization was conducted in prehybridization buffer (5 X SSC, 0.1% sodium lauroylsarcosine, 0.02% sodium dodecyl sulfate (SDS), and 1% blocking reagent (Roche) for 6 h at 42 C according to the manufacturer’s instructions (Roche). Digoxigenin (DIG)-labelled DNA probes were prepared with the PCR DIG-probe synthesis kit (Roche). Strand-specific probes were generated using the M13 primers (M13F 5′CGCCAGGGTTTTCCCAGTCACGAC3′ and M13R 5′TCACACAGGAAACAGCTATGAC3′) and appropriately digested vectors to terminate the probes at the end of inserted region. Both (+) strand specific probe and (−) strand specific probes were prepared for each cloned dsRNA. Hybridization was conducted overnight at 42°C with approximately 20 ng of probe per ml of hybridization buffer. Blots were washed twice in wash solution 1 (2 X SSC and 0.1% SDS) for 15 min each at room temperature, and then twice in washing solution 2 (0.5 X SSC and 0.1% SDS) at 68 C for 15 min each. Detection was performed by autoradiography using Lumi-Film Chemiluminescent detection film (Roche), according to the manufacturer’s instructions.

### Phylogenetic analysis

Phylogenetic analyses were separately performed for the RdRp polypeptide sequences exclusive to *O. novo-ulmi* and for the entire set of known mitovirus RdRp sequences using the Phylogeny.fr platform [41]. Sequences were aligned with MUSCLE (v3.7) configured for highest accuracy (MUSCLE with default settings; maximum number of iterations 16). The phylogenetic tree was reconstructed using the maximum likelihood method with the approximate Likelihood-Ratio Test (aLRT) implemented in the PhyML program (v3.0 aLRT) using the Whelan and Goldman (WAG) substitution model. Reliability for internal branch was assessed using the aLRT test (SH-Like). Graphical representation and editing of the phylogenetic trees were performed with TreeDyn (v198.3).

## Abbreviations

DED: Dutch elm disease; ssRNA: Single stranded RNA; dsRNA: Double stranded RNA; SPAT: Single primer amplification technique; FLAC: Full-length amplification of cDNA; cDNA: Complementary DNA; RdRp: RNA dependent RNA polymerase; vc: Vegetative compatibility; RACE: Rapid Amplification of cDNA Ends; UTR: Untranslated region; ORF: Open reading frame; BLAST: Basic Local Alignment Search Tool; RT-PCR: Reverse transcriptase PCR; PCR: Polymerase chain reaction; EDTA: Ethylenediaminetetraacetic acid; OCM: *Ophiostoma* complete medium; TAE: Tris-acetate-EDTA; HEPES: 4-(2-hydroxyethyl)-1-piperazineethanesulfonic acid; BSA: Bovine serum albumin; DTT: Dithiothreitol; DMSO: Dimethyl sulfoxide; PEG: Polyethylene glycol; SDS: Sodium dodecyl sulfate; DIG: Digoxigenin.

## Competing interests

There are no financial nor non-financial competing interests for any of the authors.

## Authors’ contributions

The manuscript was prepared by WH in consultation with JC, IK and DJ. All authors contributed to the conception and design of the experiments. Under the direct supervision of DJ and AV, IK was primarily responsible for cloning and sequencing OnuMV1c while JC cloned and sequenced OnuMV7, RNA 03 and RNA04. This study was conceived by WH and DJ. All authors have read and approved the final manuscript.
